# The effects of foot reflexology on symptoms of discomfort in palliative care: a feasibility study

**DOI:** 10.1186/s12906-023-03873-5

**Published:** 2023-02-28

**Authors:** Marie Lavarelo Marcolin, Andréa Tarot, Véronique Lombardo, Bruno Pereira, Axelle Van Lander, Virginie Guastella

**Affiliations:** 1 Université de Clermont Auvergne, CHU Clermont-Ferrand, Palliative Care Center, 63001 Clermont-Ferrand, France; 2 Université de Clermont Auvergne, CHU Clermont-Ferrand, ACCePPT UCA, Palliative Care Center, 63001 Clermont-Ferrand, France; 3 Université de Clermont Auvergne, CHU Clermont-Ferrand, Secteur Biométrie Et Médico-Économie, 63001 Clermont-Ferrand, France; 4 Palliative Care Center, CHU Clermont-Ferrand, Clermont-Ferrand, France; 5 Université de Clermont Auvergne, CHU Clermont-Ferrand, Inserm, Neuro-Dol, 63001 Clermont-Ferrand, France

**Keywords:** Foot reflexology, Massage therapy, Palliative care, Sleep quality, Anxiety, Pain sleep disorders

## Abstract

**Background:**

In palliative care, the relief of discomfort is sought by an overall approach, combining prescribed medication and additional therapies, such as foot reflexology (FR). The main objective of this study was to assess the feasibility of FR in a population of inpatients in a palliative care unit (PCU).The precariousness of the patients led us to perform a feasibility study and not a cohort study from the outset. Its secondary objective was to assess the impact of an FR session on some symptoms of discomfort (anxiety, pain, troubled sleep, and psychological distress).

**Methods:**

This is a feasibility study designed as a randomized controlled two-arm therapeutic trial. One arm tested FR, the other an active control, massage therapy (MT). The evaluators were blinded.

**Results:**

FR was feasible for 14 patients out of the 15 included in the FR group (95% CI [68%; 100%]). These patients were in the palliative care phase of cancer, motor neuron disease, or terminal organ failure. Concerning the symptoms of discomfort, ESAS sleep quality score was on average 3.9 (± 2.5) before a session in the FR group. It was improved to an average of 3 (± 2.3) on the day after the session (effect-size = 0.38 [0.03; 0.73]).

**Conclusion:**

This study confirms the feasibility of an FR session for patients hospitalized in a PCU. It resulted in a slight improvement in sleep quality. For other discomfort symptoms such as anxiety, pain and distress, FR yielded a non-significant improvement. Significant results would have needed a larger cohort.

## Introduction

Palliative care seeks to improve quality of life by relieving discomfort through pharmacological or other treatments. Among the symptoms of discomfort frequently encountered in a palliative care unit (PCU) are anxiety, pain, sleep disorders, and psychological distress. Anxiety, assessed on the Edmonton Symptom Assessment System (ESAS) scale with a median of 4/10 [1], concerns 25–30% of PCU patients [2]. Sleep disturbance and pain affect half of palliative cancer patients [3, 4]. In a study conducted in 2019, psychological distress also concerned 80% of patients, with an average score of 3.8/10 ± 2.8 [5]. The management of these symptoms is based on comprehensive multidisciplinary care. The therapies used, most often anxiolytics and opioid analgesics [6], can produce side effects such as confusion and drowsiness that impact on the patient's quality of life and relationships with their family. We looked for non-drug approaches to improve these symptoms. Foot reflexology (FR) is one such non-iatrogenic addition to drug therapy.

FR consists of a massage of specific points on the foot, aimed at stimulating reflex arcs (hence the term "reflexology"), each zone corresponding to a specific organ [7, 8]. FR commonly exerts pressure on specific areas of the foot. These areas (so-called reflexes) are assumed to correspond to a specific body part or organ, which in theory allows the practitioner to remotely stimulate the functions of these organs and regulate the energy circulation. This practice aims to relax and restore homeostasis. In some randomized trials, FR has been shown to be effective in reducing anxiety and pain and in improving sleep quality in cancer patients [9–11]. The results obtained were encouraging and significant, but their power was low [12, 13]. Many publications report on the use of FRin obstetrics [14, 15], surgery [16], cardiology [17, 18], pediatrics [19, 20], hematology [21], radiology [22], and orthopedics [23], with significant results. However, there were none on palliative care. The primary objective of this study was accordingly to evaluate the feasibility of FR in a population of patients hospitalized in a PCU. Its secondary objective was to study the effectiveness of a FR session on the relief of discomfort symptoms such as anxiety, pain, poor sleep quality, and psychological distress. Drug response was also evaluated. Moreover, the precariousness of the patients in palliative situation led us to perform a feasibility study instead of a cohort study because we could not know if patients could keep the same position all treatment long and if they could tolerate the treatment anymore.

## Methods

### Study design

This is a feasibility study designed as a randomized, controlled, two-arm therapeutic trial and its reporting is based on the Consolidated Standards of Reporting Trials (CONSORT) 2010: extension to randomized pilot and feasibility trials. Eligible patients were randomly assigned in a 1:1 ratio to foot reflexology group or active control massage therapy, using a password-protected web-based randomization system and randomization code based on computer-generated through randomly permuted blocks. One arm tested FR as a non-invasive physical intervention. FR is a systematic intervention in which applying some pressure to any particular points on the feet and hands give impacts on the health of related parts of the body (stimulation of reflex zones corresponding to specific areas of the body [7, 8]). The other arm tested an active control, namely massage therapy MT. MT [24] takes shape through touch and a sequence of movements on all or part of the body, that allows relaxation, fitness, reassurance, communication or simply well-being". The study was approved by the CPP (personal protection committee) Sud Ouest et Outre Mer III, Decembre 16, 2020. The number of the Clinical Trials registration is NCT04561271, 23/09/2020. The study took place in the PCU of the Clermont-Ferrand University Hospital. Patients were included between January 2021 and June 2021.

### Sample and measures

#### Selection of patients

We included adult patients, hospitalized in the PCU, with an alertness score ranging from + 1 to − 3 on the Richmond scale [25, 26], able to give their informed consent to take part in the research and affiliated to a social security scheme. Patients were recruited on arrival, consecutively. There were no refusals and consent was obtained within the first 24 h.

Patients with painful bone metastases in the feet, plantar pressure sores, symptomatic distal peripheral neuropathy of the lower limbs, amputation of one or both feet, impaired alertness, and patients who refused to participate, were confused, or under legal protection were not eligible for inclusion.

#### Study protocol

**Fig. 1:**
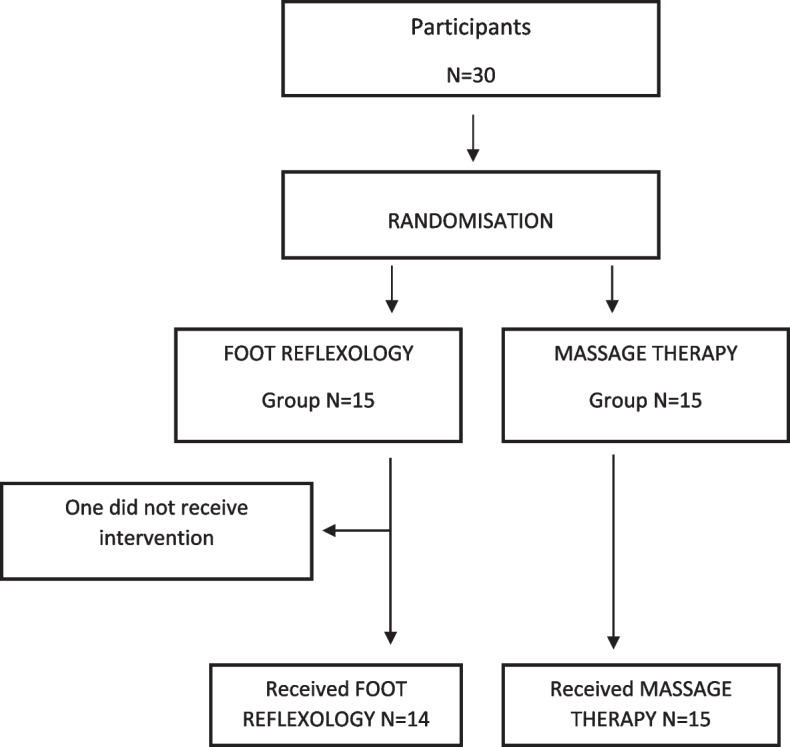
Flowchart.

Patients were presented with the study protocol, allowed a 24-h decision period, and given an information and consent form they had to sign. Patients who agreed to take part in the study were randomized to FR or MT. Both treatments involved the foot only, to maintain patient blindness until the end of the protocol. Patients in the MT group were then offered an out-of-protocol FR session. The flowchart is shown in Fig. 1.

The session was performed by a caregiver trained in FR and MT. The patient was made comfortable, either lying on their back, in a half-sitting position in bed, or sitting in a chair. The procedure lasted 15–20 min, was performed with sweet almond oil and was accompanied by soft music. The treatment was a method with sliding movements on the reflex zones. It was applied in two phases: a relaxation phase where the areas corresponding to the diaphragm and the rib cage were worked on, followed by a more specific phase where anxiety, pain and sleep quality were worked on according to the patient's needs (areas of the brain, intestines, musculoskeletal system, sinuses, solar plexus, urinary tract, and lungs), and ending with a further relaxation phase.

The survey was carried out by means of questionnaires, filled in by the patient either alone or with the help of a member of the team, 24 h before the session, just before the session, and 24 h after the session. Consumption of analgesics and anxiolytics was recorded 24 h before and 24 h after the session (the survey recorded the consumption or not of "on-demand" treatments, present in advance prescriptions: benzodiazepines, neuroleptics, paracetamol, opioids, and co-analgesics).

#### Measurements

The primary endpoint was the feasibility of a FR session for a palliative care patient in the PCU. Feasibility was defined by the conduct of a session in satisfactory conditions, i.e., for a duration of 15–20 min and a patient position maintained throughout, whether lying supine, half-sitting or sitting.

The secondary endpoints assessed symptoms of discomfort (anxiety, pain, poor sleep quality, distress). Anxiety, pain and sleep quality were assessed using the ESAS scale. This is a validated palliative care scale [27] with scores ranging from 0 (symptom absent) to 10 (symptom at worst possible severity). A change of 1 point or more was considered significant [28]. Distress was defined in cancer care in 2003, based on the work of an interdisciplinary group, as "an unpleasant experience of an emotional, psychological or spiritual nature that interferes with the ability to manage one's treatment. It extends along a continuum from feelings of vulnerability, sadness, fears, to more serious issues such as anxiety, panic attacks, depression or identity crisis [29, 30]. It is assessed by the Distress Thermometer (DT), a visual analogue scale that asks the patient to rate their feelings on a continuum from "no distress, 0" to "extreme distress, 10". The French version is validated and used in palliative care [31]. The study evaluators were blinded. The data were collected using the RedCap software. They were age, sex, occupational category, whether or not death had occurred at the time of analysis, involvement of close relatives in accompanying the patient, pathology, patient's knowledge of the diagnosis and prognosis, ESAS anxiety/pain/sleep, distress state measured by the DT, status with regard to COVID (patient and close family), state of alertness at the time of treatment (measured on the Richmond scale), use of analgesics and anxiolytics, position during treatment, duration of treatment, and whether or not a further session was scheduled.

### Statistical analysis

Because this was a pilot feasibility study, sample size estimation was based on Fleming's multi-stage design [32]. This experimental design considering a single group and sequential analyses enables a study of the feasibility set by the dichotomous criterion (more precisely its confidence interval [CI]) defined as a patient’s session going to completion (full duration and technical attainment: success defined by the performance of a session lasting for 15–20 min, the patient being able to lie supine or stay in a half-sitting or sitting position). In the light of our recruitment capacity and the literature, a two-stage design was taken, with a lower bound of maximum non-feasibility at 50% and an upper bound of minimum feasibility at 75%, with type I error and statistical power at 0.05 and 0.85 respectively. Under these conditions, we needed to include 15 patients and 13 patients respectively. Depending on these results, the decision would be made to continue or withdraw inclusions by acceptance of feasibility if the upper limit of the confidence interval of the rate of patients presenting the primary endpoint was exceeded, or by rejection if was below the lower limit of this confidence interval. At the end of the first stage, 30 patients were included (15 in the reflexology group, 15 in a control group). If 12 or more patients out of the 15 presented the primary endpoint, then the study could be stopped and the intervention considered feasible. If 8 or more patients out of the 15 failed to present the primary endpoint, then the study could be stopped for non-feasibility.

Data were recorded anonymously with REDCap software [23]. Data storage and management were performed according to international guidelines relevant in French institutions. All data were entered using electronic case report form and data accuracy was analyzed by data manager. Data quality control measures were included queries to identify outliers and missing data. Only the research assistant and study principal investigator (PI) were access to protected personal health information. After inclusion, a unique identifier (linked to the participant’s medical record number and a hard copy roster) was stored in a locked cabinet in the PI’s locked private office). The PI ensured that the anonymity is preserved. The study PI had accessed to the final trial data set, as will the biostatistician.

Continuous variables were expressed as mean and standard deviation or the median and interquartile range, according to their statistical distribution. The assumption of normal distribution was tested by the Shapiro–Wilk test. Comparisons between randomization groups for quantitative variables (symptoms changes between values at day of intervention and those 24 h afterwards) were performed with Student's *t* test or the Mann–Whitney test when conditions for the *t*-test were not met. Homoscedasticity (equality of variances) was analyzed by the Fisher-Snedecor test. The comparisons concerning categorical variables were performed with chi-squared or Fisher's exact tests. Paired comparisons (within randomization group analyses) were conducted with the paired Student test or the Wilcoxon test. As the primary objective of this study was feasibility, primary analysis was carrie out in a per-protocol population. However, a sensitivity analysis was carried out in the intention-to-treat population with a last-observation-carried-forward imputation approach. All analyses were generated with Stata software, version 15.0 (StataCorp, College Station, US). A two-sided *p* value of less than 0.05 was taken to indicate statistical significance. Results were also expressed as standardised median differences (SMD for between-group differences) and effect-size (ES for within group differences) with 95% confidence intervals. No correction for multiple testing was applied in the analysis of secondary outcomes or subgroup analysis. Because of the potential for type 1 error due to multiple comparisons, findings from analyses of secondary endpoints were interpreted as exploratory.

## Results

The study took place at the PCU of the Clermont-Ferrand Teaching Hospital. The inclusion period was from January 2021 to June 2021. 30 patients were included: 15 in the FR group, 15 in the MTgroup.

Concerning the health status of the included patients, 24 (80%) had cancer (11 (73.3%) in the FR group, 13 (86.7%) in the MT group). Detail data are reported in Table 1.The cancers included 9 lung, 4 colonic, 3 pancreatic, 2 ovarian, 2 gastric, 2 renal, 1 breast, and 1 bone. The metastatic lesions were in lymph nodes in 11 of the included patients. 11 patients had bone metastases, 9 had lung or pleural metastases, 7 had liver metastases, and 7 had peritoneal metastases. 6 patients had brain metastases. 2 patients had adrenal metastases. For the non-neoplastic diseases, 4 patients (13.3%) had a neurodegenerative disease such as motor neuron disease (2 (13.3%) in each group). The remaining 2 patients in the RP group had end-stage kidney failure in one case and mesenteric infarction in the other. Finally, 4 (13.3%) patients had end-stage organ failure (2 (13.3%) in each group). The patients included were all negative for COVID-19. This information was not always available for the patients' relatives.

**Table 1:** Baseline comparison between randomization groups, Foot Reflexology FR and Massage Therapy MT

	**Foot reflexology** ***n* = 15**	**Massage Therapy** ***n* = 15**	**All** ***n* = 30**
Sex female, *n* (%)	10 (66.7)	10 (66.7)	20 (66.7)
Age (mean ± sd)	63.7 ± 15.1	63.8 ± 14.8)	63.8 ± 14.7
(minimum – maximum)	40–97	34–85	34–97
Family status			
Married	8 (40.0)	9 (53.0)	14 (46.7)
Widowed	4 (26.6)	4 (26.6)	8 (26.7)
Single	3 (20.0)	2 (13.3)	5 (16.7)
Divorced	1 (6.7)	1 (6.7)	2 (6.7)
Partner	1 (6.7)	0 (0.0)	1 (3.3)
Occupational status			
Retired	10 (66.7)	8 (53.3)	18 (60.0)
Active	4 (26.7)	4 (26.7)	8 (26.7)
Unemployed/unknown	1 (6.7)	3 (20.0)	4 (13.3)
Occupation			
Artisan	1 (9.1)	2 (20.0)	3 (14.3)
Trader	1 (9.1)	0 (0.0)	1 (4.7)
Manager, higher intellectual	4 (36.4)	0 (0.0)	4 (19.0)
Intermediate	0 (0.0)	3 (30.0)	3 (14.3)
Employee	5 (45.4)	5 (50.0)	10 (47.6)
Disease			
Cancer	11 (73.3)	13 (86.7)	24 (80.0)
Neurodegenerative disease	2 (13.3)	2 (13.3)	4 (13.3)
Organ failure	2 (13.3)	2 (13.3)	4 (13.3)
Relative presence (number of patients)	15 (100)	14 (93,3)	29 (96.7)
Patients informed of diagnosis	15 (100)	15 (100)	30 (100)
Patients informed of prognosis	15 (100)	15 (100)	30 (100)
Palliative phase	15 (100)	12 (80.0)	27 (90.0)
COVID 19 infection	0 (0.0)	0 (0.0)	0 (0.0)
Death	13 (86.7)	11 (78.6)	24 (82.7)
Richmond Scale 0 value *n* (%)	13 (86.7)	11 (73.3)	24 (80.0)
Anxiety	3.3 ± 2.1	3.3 ± 3.4	3.3 ± 2.8
Pain	3.6 ± 2.2	2.8 ± 1.5	3.2 ± 1.9
Sleep quality	4.3 ± 2.1	4.6 ± 2.9	4.4 ± 2.5
Distress	3.2 ± 1.6	3.4 ± 2.8	3.3 ± 2.2

FR was feasible for 14 of the 15 patients included (95% CI [68%; 100%]). The patients were terminally ill, with cancer, motor neuron disease, or end-stage organ failure, and had no cognitive impairment or confusion. All were able to maintain the same position throughout the session. Of the 30 patients, half chose the supine position (8 (53.3%) in the FR group, 7 (46.7%) in the MT group), the other half the half-sitting position (7 (46.7%) in the FR group, 8 (53.3%) in the MT group). Of the patients included in the study, 17 (60.71%) received a second session.

For discomfort symptoms, there was no statistically significant difference between the randomization groups for sleep (SMD =  − 1 [− 2.5; 0.5]), anxiety (SMD = 1 [− 0.5, 2.0]) or pain (SMD = 1 [− 1.0; 3.0]). However, for anxiety and sleep, the change was statistically different between the day of the intervention and the 24 h assessment for sleep (ES = 0.38 [0.03; 0.73]) and for anxiety (ES = 0.47 [0.02; 0.91]) for the FR group. For psychological distress, the variation was statistically different between the randomization groups (SMD = 1 [0.0; − 2.5]). The results are presented in Table 2. Sensitivity analysis conducted in the intention-to-treat population highlighted analoguous results.

**Table 2:** Difference between discomfort symptoms the day and 24 h after Foot Reflexology or Massage Therapy

	**Foot reflexology *n* = 14**		**Massage Therapy *n* = 15**		**Standardized median difference (95%CI) * p*-value**
	**Row data Mean ± sd**	**Change med [IQR]**	**Row data Mean ± sd**	**Changemed [IQR]**	
Sleep, *mean* ± *standard-deviation*					
Day of intervention	3.9 ± 2.5		5.4 ± 3.0		
24 h after intervention	3.0 ± 2.3	− 1 [− 2; 0]*	4.9 ± 3.1	0 [− 2; 0]	− 1 [− 2.5; 0.5], *p* = 0.617
Distress, *mean* ± *standard-deviation*					
Day of intervention	2.5 ± 1.9		3.4 ± 3.3		
24 h afterwards	2.5 ± 1.9	0 [− 1; 1]	2.2 ± 2.9	− 1 [− 2; 0]*	1 [0.0; − 2.5], *p* = 0.049
Anxiety, *mean* ± *standard-deviation*					
Day of intervention	3.1 ± 2.0		3.2 ± 3.5		
24 h afterwards	2.2 ± 1.7	0 [− 2; 0] *	2.2 ± 2.4	− 1 [− 2; 0]	1 [− 0.5, 2.0], *p* = 0.855
Pain, *mean* ± *standard-deviation*					
Day of intervention	3.1 ± 1.2		2.7 ± 1.5		
24 h afterwards	2.7 ± 1.8	0 [− 1; 1]	1.7 ± 1.7	− 2 [− 1; 0]	1 [− 1.0; 3.0], *p* = 0.314
Antalgics, *n* (%)					
Day of intervention	6 (40.0)		4 (26.7)		
24 h afterwards	5 (33.3)		4 (26.7)		
Anxiolytics, *n* (%)					
Day of intervention	3 (20.0)		2 (13.3)		
24 h afterwards	3 (20.0)		2 (13.3)		

^*^indicates *p* < 0.05 between measures at day of intervention and 24 h after intervention (within randomization groups)

Concerning the consumption of analgesics, the difference before and after a session was not significant (*p* = 1 in the FR group, *p* = 0.6 in the MT group), and there was no significant difference between the two groups (33.3% vs. 26.7%) after the intervention. Consumption of weak opioids, paracetamol, and coadministrative analgesics on demand was low. The difference in benzodiazepine and neuroleptic consumption before and after a session was not significant (*p* = 1 for each drug class, in the MT and FR groups).

## Discussion

The results of this study confirm the feasibility of an FR session for palliative care inpatients. This patient profile is found in other studies evaluating the efficacy of non-pharmacological treatment in a PCU, including the Schubert dressing [33]. The length of the session and the need to maintain the same position throughout led us to expect poor tolerance and discomfort. However, of the 30 patients included, only one (3.33%), belonging to the FR group, was unable to complete a session, which was shortened to less than 15 min owing to confusion.

Just over half of the patients [17] received a second session, outside the protocol. This supports possible repeatability of the treatment. The reasons for not having an additional session were deterioration of the patient's clinical condition, impossibility of rescheduling the session, or a patient not wanting a new session, for reasons which were not recorded.

Our study has some bias. Although confusion was one of the non-inclusion criteria of the study, a patient could have developed confusion between the time of inclusion and the self-assessment of their symptoms. Confusion is a common symptom in palliative care situations, especially terminal ones.

The FR session did not lead to any increase in the intensity of the discomfort symptoms. In most cases it reduced them. It was in the improvement of sleep quality that the results were significant. After an FR session, the ESAS score was improved by 1.1 points. The day after the session, patients often spontaneously reported to the doctor or carers that the treatment had helped them sleep better. For the other symptoms, inclusion of a larger number of patients would have been needed to yield more significant results. FR could not be shown to be drug-sparing in the management of anxiety and pain, probably because of too few patients in each subgroup, the complexity of their profiles, and the many factors outside the session that influenced their health status. No significant difference was found compared with MT except for the symptom of distress. MT reduced distress more significantly. This result can be explained by the quality of the touch massage, which was provided in a care relationship dealing with distress and care oriented toward well-being.

No other feasibility study of FR on palliative care patients is described in the published literature. Altough, this study confirms the feasibility of an FR session for patients hospitalized in a PCU, it did not result in a statistically significant improvement in sleep quality. For other discomfort symptoms such as anxiety, pain and distress, FR yielded a non-significant improvement. We are currently including more patients to support our results, which could also be strengthened by qualitative analysis. These techniques are consistent with the overall care provided in a PCU, i.e., intended to improve the quality of remaining lifespan.

## Data Availability

Data and all of the material are owned by the authors. The data that support the findings of this survey are available from the corresponding author upon reasonable request.

## References

[1] Follwell M, Burman D, Le LW, Wakimoto K, Seccareccia D, Bryson J (2009). Phase ii study of an outpatient palliative care intervention in patients with metastatic cancer. J Clin Oncol.

[2] Temel JS, Greer JA, Muzikansky A, Gallagher ER, Admane S, Jackson VA (2010). early palliative care for patients with metastatic non–small-cell lung cancer. N Engl J Med.

[3] Nzwalo I, Aboim MA, Joaquim N, Marreiros A, Nzwalo H (2020). Systematic review of the prevalence, predictors, and treatment of insomnia in palliative care. Am J Hosp Palliat Med.

[4] Kaasa S, Loge JH, Aapro M, Albreht T, Anderson R, Bruera E (2018). Integration of oncology and palliative care: a lancet oncology commission. Lancet Oncol.

[5] Van Lander A, Tarot A, Savanovitch C, Pereira B, Vennat B, Guastella V (2019). Assessing the validity of the clinician-rated distress thermometer in palliative care. BMC Palliat Care.

[6] Lycken M, Drevin L, Garmo H, Stattin P, Adolfsson J, Lissbrant IF (1990). The use of palliative medications before death from prostate cancer: Swedish population-based study with a comparative overview of European data. Eur J Cancer Oxf Engl.

[7] Abbaszadeh Y, Allahbakhshian A, Seyyedrasooli A, Sarbakhsh P, Goljarian S, Safaei N (2018). Effects of foot reflexology on anxiety and physiological parameters in patients undergoing coronary artery bypass graft surgery: A clinical trial. Complement Ther Clin Pract mai.

[8] Wyatt G, Sikorskii A, Tesnjak I, Frambes D, Holmstrom A, Luo Z (2017). A randomized clinical trial of caregiver-delivered reflexology for symptom management during breast cancer treatment. J Pain Symptom Manage.

[9] Tsay SL, Chen HL, Chen SC, Lin HR, Lin KC (2008). Effects of reflexotherapy on acute postoperative pain and anxiety among patients with digestive cancer. Cancer Nurs avr.

[10] Stephenson NL, Weinrich SP, Tavakoli AS (2000). The effects of foot reflexology on anxiety and pain in patients with breast and lung cancer. Oncol Nurs Forum févr.

[11] Quattrin R, Zanini A, Buchini S, Turello D, Annunziata MA, Vidotti C (2006). Use of reflexology foot massage to reduce anxiety in hospitalized cancer patients in chemotherapy treatment: methodology and outcomes. J Nurs Manag mars.

[12] Deng G, Cassileth B (2013). Complementary or alternative medicine in cancer care-myths and realities. Nat Rev Clin Oncol.

[13] Cassileth BR (2014). Psychiatric benefits of integrative therapies in patients with cancer. Int Rev Psychiatry Abingdon Engl févr.

[14] Arnon Z, Dor A, Bazak H, Attias S, Sagi S, Balachsan S, et al. Complementary medicine for laboring women: a qualitative study of the effects of reflexology. J Complement Integr Med 26 mars 2019;16(1). Disponible sur: http://www.degruyter.com/view/j/jcim.2019.16.issue-1/jcim-2018-0022/jcim-2018-0022.xml [Cité 15 déc 2019] 10.1515/jcim-2018-0022 30024855

[15] YılarErkek Z, Aktas S (2018). The effect of foot reflexology on the anxiety levels of women in labor. J Altern Complement Med N Y N.

[16] Öztürk R, Sevil Ü, Sargin A, Yücebilgin MS (2018). The effects of reflexology on anxiety and pain in patients after abdominal hysterectomy: A randomised controlled trial. Complement Ther Med févr.

[17] Bahrami T, Rejeh N, Heravi-Karimooi M, Tadrisi SD, Vaismoradi M (2019). The effect of foot reflexology on hospital anxiety and depression in female older adults: a randomized controlled trial. Int J Ther Massage Bodyw sept.

[18] Ramezanibadr F, Amini K, Hossaingholipor K, Faghihzadeh S (2018). The impacts of foot reflexology on anxiety among male candidates for coronary angiography: A three-group single-blind randomized clinical trial. Complement Ther Clin Pract août.

[19] Bertrand A, Mauger-Vauglin CE, Martin S, Goy F, Delafosse C, Marec-Berard P (2019). Evaluation of efficacy and feasibility of foot reflexology in children experiencing chronic or persistent pain. Bull Cancer (Paris).

[20] Canbulat Sahiner N, Demirgoz BM (2017). A Randomized Controlled Trial Examining the Effects of Reflexology on Children With Functional Constipation. Gastroenterol Nurs Off J Soc Gastroenterol Nurses Assoc.

[21] Rambod M, Pasyar N, Shamsadini M (2019). The effect of foot reflexology on fatigue, pain, and sleep quality in lymphoma patients: a clinical trial. Eur J Oncol Nurs.

[22] Parmar R, Brewer BB, Szalacha LA (2018). Foot massage touch, and presence in decreasing anxiety during a magnetic resonance imaging a feasibility study. J Altern Complement Med N Y N.

[23] Pasyar N, Rambod M, Kahkhaee FR (2018). The effect of foot massage on pain intensity and anxiety in patients having undergone a tibial shaft fracture surgery: a randomized clinical trial. J Orthop Trauma déc.

[24] Kutner JS, Smith MC, Corbin L, Hemphill L, Bento K, MellisBeaty BKB, Felton S, Yamashita TE (2008). massage therapy vs simple touch to improve pain and mood in patients with advanced cancer a randomized trial. Ann Intern Med.

[25] Sessler CN, Gosnell MS, Grap MJ, Brophy GM, O’Neal PV, Keane KA (2002). The richmond agitation-sedation scale: validity and reliability in adult intensive care unit patients. Am J Respir Crit Care Med.

[26] Chanques G, Jaber S, Barbotte E, Verdier R, Henriette K, Lefrant JY (2006). Validation de l’échelle de vigilance–agitation de Richmond traduite en langue française. Ann Fr Anesth Réanimation.

[27] Bruera E, Kuehn N, Miller MJ, Selmser P, Macmillan K (1991). The Edmonton symptom assessment system (esas): a simple method for the assessment of palliative care patients. J Palliat Care juin.

[28] Hui D, Shamieh O, Paiva CE, Perez-Cruz PE, Kwon JH, Muckaden MA (2015). Minimal clinically important differences in the Edmonton symptom assessment scale in cancer patients: a prospective, multicenter study. Cancer.

[29] Van Lander A. L'identité à l'épreuve de la maladie létale. Psychologie. Université Lumière Lyon II, 2012. Français IdRef : 171331796.

[30] Van Lander A. Crise d’identité du Mourir vol. 2ème ed. dunod; 2017 disponible sur: https://www.cairn.info/soins-palliatifs--9782100759910-page-231.htm [cité 19 août 2021].

[31] Tarot A, Van Lander A, Pereira B, Guastella V (2019). Étude de la relation entre détresse et confusion mentale chez les patients en situation palliative (Relation entre détresse et confusion mentale en situation palliative). Médecine Palliat déc.

[32] Ratain MJ, Sargent DJ (2009). Optimising the design of phase II oncology trials: The importance of randomisation. Eur J Cancer janv.

[33] Oppert C, Gomas JM. Analyse de l’impact des séances d’art-thérapie musicale « Pansement Schubert ». J Pain Symptom Manage déc. 2018;56(6):e50–1.

